# Falling and rising in the vortex of cancer: children’s adaptation with cancer: a qualitative study

**DOI:** 10.1186/s40359-024-01722-9

**Published:** 2024-04-22

**Authors:** Fatemeh Sepahvand, Fatemeh Valizadeh, Kimia Karami, Babak Abdolkarimi, Fatemeh Ghasemi

**Affiliations:** 1 Student Research Committee, Lorestan University of Medical Sciences, Khorramabad, Iran; 2 Social Determinants of Health Research Center, Lorestan University of Medical Sciences, Khorramabad, Iran; 3 Pediatrics Oncologist-Hematologist, Lorestan University of Medical Sciences, Khorramabad, Iran; 4 Department of Pediatrics Nursing, School of Nursing and Midwifery, Lorestan University of Medical Sciences, Khorramabad, Iran

**Keywords:** Child, Cancer, Adaptation, Nursing model, Qualitative research, Callista Roy

## Abstract

**Background:**

Cancer is a considerable health problem worldwide and the second leading cause of death in children. It has many physical, psychological, and social consequences for children and their families. The ability to adapt to cancer plays a vital role in the recovery and quality of life of affected children. This study aimed to explain the adaptation of children with cancer to their disease.

**Methods:**

This qualitative study adopted the directed content analysis approach based on the Roy nursing model. The participants were nine children with cancer aged 6–18 years old, five family members, four nurses, one doctor, one teacher, and two charity association members, recruited by purposive sampling method. The information was collected via individual semi-structured interviews, a focus group discussion, and field notes. The data were analyzed simultaneously with data collection using the Elo and Kyngäs method. The study rigor was ensured based on the Guba and Lincoln criteria.

**Findings:**

Of the four categories of physical challenges, fragile self-concept, the difficulty of role transition, and disruption of the path to independence, the theme of Falling and rising in the cancer vortex was abstracted.

**Conclusion:**

Based on the Roy model, the children in the present study were at the compensatory level of adaptation. This research demonstrates that the adaptation of children being treated for cancer is fragile and not constant. With each hospitalization and exacerbation of the disease, they made efforts to adapt to their disease using regulatory and cognitive subsystems. Paying attention to different stimulants and the effects of support systems on physical challenges, fragile self-concept, difficult role transition, and disruption of the path to independence for each child, as well as providing individualized care for these children, can help their adaptation to and healthy transition from the vortex of cancer. The Roy adaptation model was helpful and efficient for elucidating the adaptation of children with cancer. Providing care for children by healthcare specialists, especially nurses, should be theory-based and individualized.

**Supplementary Information:**

The online version contains supplementary material available at 10.1186/s40359-024-01722-9.

## Background

Cancer is a major health problem and its increasing growth in recent decades, along with its negative impacts on physical, psychological, social, and economic aspects of human life, have greatly concerned experts [1]. Nowadays, with the development of medicine, cancer is gradually transforming from an acute and fatal disease into a chronic one in children and adolescents. Despite the increased survival rate, cancer causes life-threatening conditions [2]. Children and their families often report psychosocial stressors of cancer, such as loss of control over emotions, fear, distancing from the family, disrupted family practices, family conflicts, financial problems, and loss of social relationships with peers [3]. When a child is diagnosed with cancer, it can lead to psychological distress, including adaptation problems and its destructive consequences such as lack of adherence to health recommendations that is highly prevalent among the patients with chronic diseases [4, 5]. Lack of treatment adherence can continually reduce the patients’ quality of life and may even endanger their life [6]. Therefore, checking and helping to manage adaptation in children with cancer is one of the important nursing measures.

**Fig. 1:**
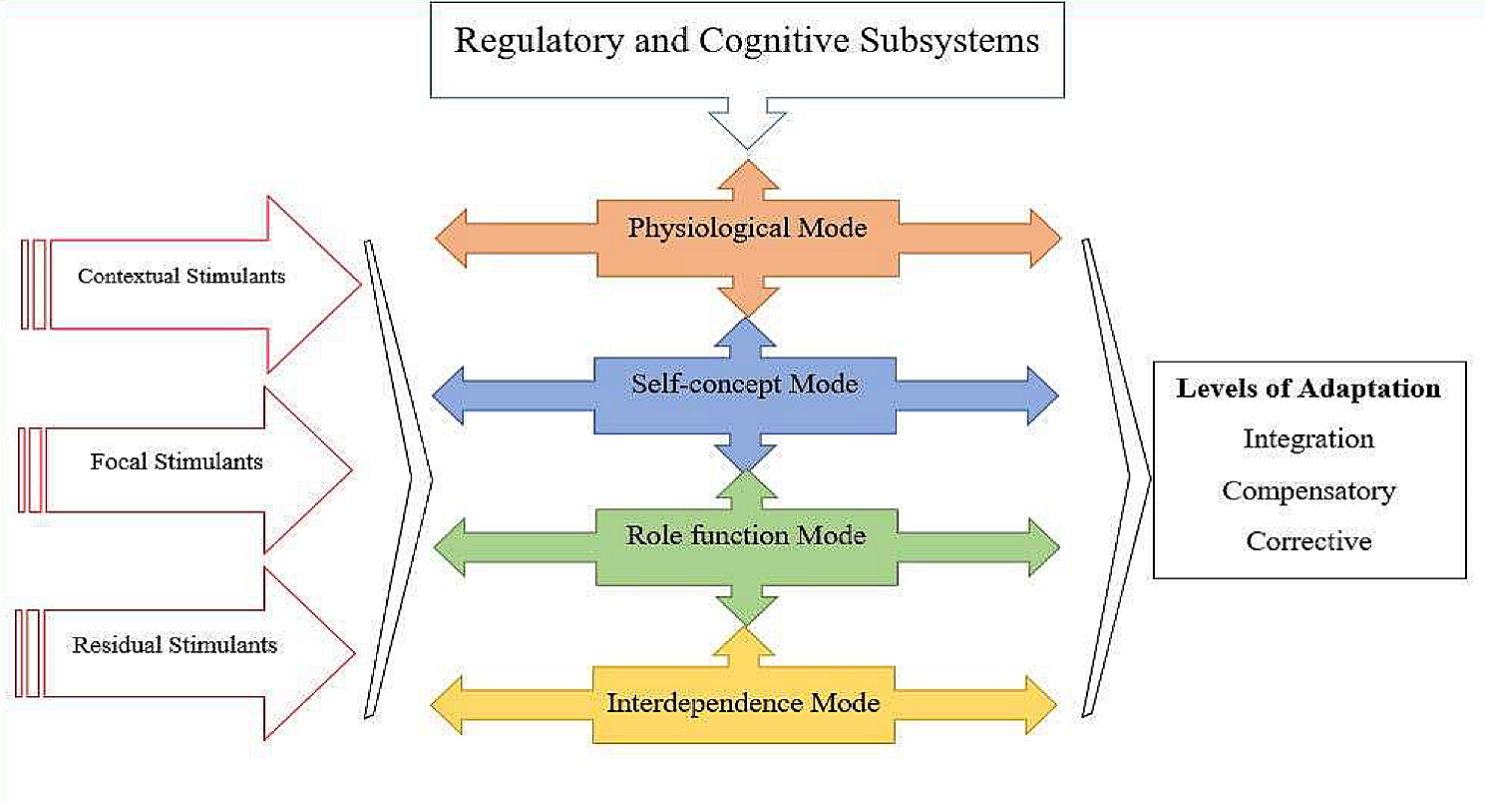
The Roy Adaptation Model (2009)

Nursing theories and conceptual models regulate nurses’ activities and direct them in research, practice, education, and management [7, 8]. The Roy adaptation model (2009) (Fig. 1) views individuals and groups as adaptive comprehensive systems that are continually in relationship and interaction with the environment and its stimulants [9, 10]. Adaptive behaviors are positive responses to the focal, contextual, and residual stimulants [11]. Roy introduces two regulatory and cognitive subsystems to help the process of adaptation [12]. She has described three levels of adaptation: 1- integration level, which structures, performances, and processes are activated to meet one’s needs in life; 2- compensatory level, which the subsystems are activated to integrate the processes of one’s life; and 3- corrective level, which compensatory systems are inefficient and a negative response is formed [7]. The general goal of nursing is to focus on promoting individual or group health by increasing adaptation in four physiological, self-concept, role function, and interdependence modes [11]. Kohi et al. conducted a qualitative study to determine the concerns and needs related to cancer among young adults and children with cancer in Tanzania. They stated that more studies are required to understand and elucidate this group’s daily needs and concerns, especially in third world and low-income countries [13]. Children’s adaptation to cancer has so far been largely based on the experiences of survivors and parents. The adaptation of children with cancer during the treatment phase has been less studied based on their own experiences. Therefore, this study aimed to elucidate the adaptation of children with cancer based on the Roy adaptation model.

## Method

### Design

This was a qualitative study using directed content analysis. It was conducted in the pediatric oncology ward in Shahid Madani hospital, Khorramabad, Iran. Children with cancer in Iran are treated in 32 hospitals under the supervision of universities of medical sciences. Treatments such as surgery, chemotherapy, radiotherapy, hormone therapy, and transplantation are performed for cancer patients in big cities in Iran. Childhood cancer treatment costs are covered by special and incurable disease insurance and non-governmental organizations (NGOs) such as the Mahak charity. Mahak provides support, psychosocial and welfare services to cancer children and their families [14, 15]. Shahid Madani Hospital has a children’s oncology ward. In this ward chemotherapy and medical treatments are done for children with cancer. Patients go to Tehran and big cities for other treatments such as surgery, radiotherapy, and…. In this hospital, Hamin charity affiliated to Mahak was established in 2015. Benevolent people and volunteers cooperate with this charity. Their purpose is to entertain and sometimes help with children’s educational issues, and to a limited extent financial aid to patients who are introduced to them by the head nurse or ward oncologist. Providing the services usually is limited to underprivileged ones and the lack of a formal system for notifying the patient and family has caused some families to be unaware of these services and bear the treatment costs themselves.

### Participants

There were 22 participants, 15 females and 7 males, the main participants were nine children aged 6–18 with Acute lymphoblastic leukemia, Hodgkin’s lymphoma, or Osteosarcoma or etc.; however, based on the created concepts, other participants including five family members, four nurses, one oncologist, one teacher and two members of Hamin charity were also recruited later (appendix 1). Purposive sampling was performed from June 2019 to September 2020. Efforts were made to have maximum diversity in sampling in terms of sex, age, diagnosis, etc., so that the data would better represent the population. The sampling continued until the data reached saturation. The inclusion criteria for children were willingness to participate, have the age of 6–18 years old; 6 months have passed since their definitive diagnosis of cancer, lack of communication problems and no known history of psychological diseases. The exclusion criteria were the incidence of any events during the interview that would prevent the interview from moving on and unwillingness to continue participation.

### Data collection

The main data collection method was in-depth, semi-structured interviews. A preliminary interview guide (Box 1) based on the four modes of the Roy adaptation model was used. During the interviews, based on the analysis, the questions were gradually added to this preliminary guide. The questions were posed to the participants in the form of reminding and recounting a memory. For children, the questions were made suitable to their developmental stage. The location and time of conducting the interviews were set with the participants and the interviews were audio-recorded with their permission. Moreover, notes were taken on the gestures, pauses, and other non-verbal communications of the participants. Each interview lasted for 30–45 min based on the participants’ cooperation. In addition, for brainstorming and further encouraging the children to talk, the focus group discussion with three children was also held but because of the COVID-19 pandemic, the focus group discussion was held online. When present in the field, the researcher recorded everything observed, heard, or experienced in relation to the research topic in the form of six field notes, which served as another means of data collection.

**Box 1:** Preliminary interview guide

Interview Guide	
**Question for Physiological Mode**: *“How has your physical condition changed since you became ill? What changes has your body made since then?”* **Question Self-concept Mode**: “*How did you feel about yourself since you got sick? What did you like about yourself? What did you not like about yourself? How about now?”* **Question Role function Mode**: *“How did your illness affect the things you used to do (for example, going to school, playing games, things you did at home)? What about now? What things did you do that you did not do before? What things did you do that helped you?” “What did you do that did not help you?”* **Question Interdependence Mode**: *“Has your illness affected your relationship with family/friends/nurse/doctor or others? How now? Who helped you? For example, what to do? Who did not help you? For example, what to do?”*	

### Data Analysis

The interviews and field notes were transcribed on paper verbatim as soon as possible, then typed in Microsoft Word software and then entered into MaxQDA software version 2010. The data were analyzed simultaneously with data collection using the Elo and Kyngäs method. According to Elo and Kyngäs, content analysis comprises three main phase: preparation, organization, and reporting [16, 17]. In total, 1010 codes were obtained upon data analysis. Upon continuous comparison and placement of similar codes in a previously prepared matrix based on the Roy model, four themes of physical challenges, fragile self-concept, difficulty of role transition, and disruption of the path to independence were determined (Table 1).

### Rigor

The Guba and Lincoln trustworthiness criteria, credibility, dependability, confirmability, and transferability were used to ensure the rigor of the study [18]. To enhance the credibility of the findings, prolonged engagement with the participants and member checks (the findings of the study were seen by three participants for confirmation and they stated that they were accurate in their perception and transfer of experiences) were used. The dependability of the data was confirmed by transcribing the interviews as soon as possible, seeking peers’ opinions, and reviewing the entire data. To guarantee confirmability, a voice recorder was used to record all the interviews. In addition to the comments and participation of the research team, the comments of two external appraisers were used. In all the stages of the study, parts of the codes, subcategories, and categories were given to them, and they were requested to examine the process. Then, their comments for confirmation or modification were applied. To guarantee data transferability, sampling was performed with maximum diversity.

## Results

Experiences of children with cancer in terms of adaptation were described as falling and rising in the vortex of cancer. Based on this concept, children are constantly trying to adapt to their disease based on their disease recovery/exacerbation status and support systems.

**Table 1:** Categories, subcategories, and initial categories

**Physical challenges (physiological mode)**																													
Disruption in breathing pattern			Disruption in nutritional pattern						Disruption in activity and rest pattern							Collapse of the protective system					Unpleasant accompaniment of pain							Agitated nerves	
Oxygenation less than the body’s need	Need for respiratory support		Change in food intake level	Adherence to the diet	Lack of adherence to the diet		Weight fluctuations		Limitations in performing daily activities		Limited leisure		Change in play pattern		Disruption in sleep pattern	Disruption in skin integrity	Hair loss		Immune system weakness		Pain due to cancer	Pain due to diagnosis and treatment	Pharmacological methods of pain relief	Non-Pharmacological methods of pain relief	Inefficiency of pain relief methods			Symptoms of CNS involvement	Resolving neurological symptoms post-treatment
**Fragile self-concept (self-concept mode)**																													
Distorted body image (physical self)										Unstable personal self (personal self)																			
Feeling of ugliness following hair loss			Efforts to regain fitness							Apprehension			Emotional behavior					Fragile spirituality				Threatened future					Efforts for positive thinking		
**Difficulty of role transition (role function mode)**																													
Responsibility despite cancer (primary role)						Loss of income (secondary role)										Efforts to tolerate cancer (tertiary role)													
Adjusting responsibility at home		Continuing education despite difficulties				Employment before the disease						Quitting one’s job after the disease				In search of diagnosis				Cancer bottlenecks			Adherence to treatment			Reduction in social activities			
**Disruption of the path to independence(interdependence mode)**																													
Influential others (significant others)								Insufficient support systems																					
Empowering behaviors			Limiting behaviors					Financial problems of the family						Lack of an integrated and purposive treatment system				Sufficiency of the educational system				Helpfulness of charity associations					Lack of specialized and all-inclusive insurance		

### Physical challenges

The physical challenges experienced by these children included disruption in breathing pattern, disruption in nutritional pattern, disruption in activity and rest pattern, collapse of the protective system, unpleasant accompaniment of pain, and agitated nerves. The mother of a participant mentioned: “Every time his disease relapses, he breathes with difficulty and must receive oxygen all the time.” (Participant 4).

The field note about disruption in nutritional pattern: “The child felt nauseated and loudly gagged several times.” The mother stated: “She hasn’t even had water and her stomach is completely empty.” (Field note 3).

A 17-year-old adolescent said: “I experience weakness and malaise because of the medication. I feel dizzy”. (Participant 2)

A doctor said: “They experience hair loss due to the side-effects of the medications. Their skin is dried or discolored on the face, eyebrows, and eyelids. White lines may appear on the nails.” (Participant 20).

A 13-year-old boy said: “I was in a lot of pain. I could not sleep at night because of it. I had to take a bath and pour warm water on it to relieve pain. My mom kept massaging my feet, but the pain wouldn’t go away.” (Participant 21).

### Fragile self-concept

Based on the findings, extensive and debilitating cancer can lead to a “distorted body image” in the physical self and a “fragile personal self” in the personal self-component. “Feeling of ugliness following hair loss” and “efforts to regain fitness” were the major concepts of distorted body image.

A 10-year-old boy said, “I’m not happy about my hair loss; I feel embarrassed. I have become ugly. I was very pretty when I had hair.” (Participant 7).

The aunt of a patient mentioned: “He is really sad because he has been obese due to the medications. He hardly do exercise to lose weight with the help of his father.” (Participant 12).

“Apprehension”, “emotional behaviors”, “fragile spirituality”, “threatened future”, and “efforts for positive thinking” lead to an “unstable personal self” in children with cancer.

“Apprehension” was abstracted from “rumination of hospitalization” and “distress and torment of diagnostic-therapeutic methods”. The thought or experience of painful diagnostic and therapeutic methods made these children feel distressed and tormented constantly. A 13-year-old girl stated, “I couldn’t sleep until 5 in the morning last night because I knew I’d be hospitalized the next day. I kept thinking how many venous catheters they’d be placing.” (Participant 22).

These children demonstrated “emotional behaviors” such as “crying”, “agitation”, “impatience”, “discomfort”, and “grumpiness”. A 17-year-old boy stated, “I’ve become angry, stubborn, and cross. I do not want to use my disease as an excuse. However, it’s something others have to accept. Anyone who sits on these beds will start nagging and being stubborn.” (Participant 3).

The patients and their families took refuge in religious and cultural beliefs to control and manage the stress caused by cancer. These children and their families believe in invoking God and saints for recovery. Thus, when their patients are not recovered and the treatment is prolonged, they start complaining to God and the saints. One of the participants said: “I used to tell God, ‘Cure all the patients who are at the hospital. Then cure me too. Don’t let me suffer too much. Sometimes, I complained to Him about being ill.” (Participant 22).

Upon experiencing “lost health and beauty”, “endangered academic-occupational future”, “disrupted life routine”, and “endangered life expectancy” as well as with the prolongation of the treatment process and lack of recovery, some children lost their hope and spirits, and became depressed over time. A nurse declared the following about a 17-year-old boy: “His mother says he has thought about suicide several times.” (Participant 18).

Some children believed that maintaining their spirits affected their recovery. One of the participants said: “When this happened to me, I decided to make myself happy, instead of being sad. I keep telling myself it’s like a cold and I’ll be fine one day. I imagine the future when I’m fine.” (Participant 21).

### Difficulty of role transition

A child’s primary role in the family is that of a child and a student, and playing these roles is difficult under the effect of cancer. However, in most cases, children tried to demonstrate “responsibility despite cancer” by “adjusting responsibility at home” and “continuing education despite difficulties”. A mother mentioned: “During treatment, she kept cleaning her room; even when she was in a poor physical condition.” (Participant 22).

A 16-year-old girl stated: “At the beginning of the disease, I asked my doctor, ‘What will happen to my studies?’ He replied, ‘A teacher will attend the ward.’ When we went back home, we hired a tutor, also my elder sister and brothers helped me. I went to school only for the exams.” (Participant 1).

Loss of income (secondary role): Some adolescents used to work and earn money for themselves and their families, and the disease led to the loss of their jobs and income. A 17-year-old boy stated, “I used to be a street vendor, but, physically, I can’t do it anymore.” (Participant 5).

Trying to tolerate cancer (tertiary role) expresses the participants’ experiences in making efforts to accept the patient role. Primary categories were: “in search of diagnosis”, “cancer bottlenecks”, “treatment adherence”, and “reduction the social activities”. Many children and families ignored the primary physical symptoms and turned to “self-treatment”; this led to a “delayed visit to the doctor”. A participant mentioned: “My foot hurt so much. I took a pack of Ibuprofen a week, with no effect. Then, I told my dad, ‘Let’s go to a doctor or hospital and see what it is.’ (Participant 21).

The participants declared that, after the diagnosis of cancer, they faced “cancer bottlenecks” including efforts to understand cancer, hiding the disease from others, not accepting the limitations, and the need for the passage of time. These children were making “efforts to understand cancer” by understanding it through experience, asking others, searching the Internet, and reading books. A 17-year-old boy said, “No one has told me its cancer. I searched the Internet and found what it was. When I ask my mother, she says it’s not cancer.” (Participant 5).

Many children did not wish for others to know their diagnosis and the reason was the fear of being abandoned by them. This is why they did not wear a hat or mask because it would attract attention, and it would bother them if their family would tell others about their disease. A 13-year-old boy said, “People kept asking me why my hair was falling. I was fed up with all these questions. I was afraid they’d leave me if they’d know what my disease was.” (Participant 21).

“Lack of adherence to doctors’ orders”, “lack of adherence to isolation and “lack of adherence to dietary restrictions” were evidence for “not accepting the limitations”. A mother said, “The doctor wouldn’t allow me to send him to school, but I do because he really likes to go.” (Participant 4).

The adaptive behaviors experienced by these children included “adherence to doctors’ orders”, “performing diagnostic and therapeutic tests”, “adherence to isolation”, and “searching for a healthy lifestyle”. When the researcher was present in the field and during patient visits, a 17-year-old patient posed the following questions to his doctor: “What can I eat? Would take-out be harmful? Can I go to my friend’s birthday party? Would riding a motorbike hurt me? Can I go to the gym?” (Field note 3).

Due to the nature of the disease or the medication side effects, these children experienced a “reduction in social activities”. A participant mentioned: “Before this disease, I had a black belt in Karate. But after the disease, I had to quit it.” (Participant 5).

### Disruption of the path to independence

In this study, significant others included the family, friends, relatives, teachers, ward psychologists, nurses, and doctors.

“Others’ educational role”, “others’ dutifulness”, “boosting morale”, “normalization”, and “continuing relationships with others” were among the children’s empowering behaviors by significant others, while “a sense of debt to the family” and “entertaining oneself alone” were the children adaptive behaviors.

Children felt a debt to the family because the family took the trouble of maintaining hygiene, took care of them, felt sad because of their illness, and had financial problems because of the disease. Some children made “efforts to compensate for the family’s troubles” by thanking their mothers for always accompanying them in the hospital, improving their relationship with the family because of their support, and studying during treatment. The aunt of a participant said: “My niece used to say, ‘I should compensate for this disease and the trouble it has caused for my family by studying and education’” (Participant 12).

“Social isolation” and “emotional conflict with others” were the significant others’ limiting behaviors towards the children, whereas “envying others’ health” and “having expectations of others” were among the children’s maladaptive behaviors. A 10-year-old girl noted, “My friends laugh at me; they call me bald. I don’t like to be called bald. It offends me. I don’t play with them anymore.” (Participant 6).

A 10-year-old boy declared: “When I watch healthy children playing from the window, I feel sad because I can’t play like them” (Participant 23).

In the focus group discussion, one of the participants mentioned the following about the nurse’s crying during cannulation: “Ms.…[the nurse] has cannulated half my veins; she cried when she saw my state.” (Participant 23).

According to the participants, support systems included the family, the healthcare system, the educational system, the members of charity associations, and insurance companies. Almost all the significant others for the children were part of their support system as well. The preliminary categories of this subcategory were “the financial problems of the family”, “a lack of an integrated and purposive healthcare system”, “lack of specialized insurance”, “sufficiency of the educational system”, and “benefits of the charity associations”.

A nurse said: “We had a patient, the family of whom did not have a good economic status; he was really sad and had not coped with his disease. He was hurt more because there was also a financial burden to bear. There was once a boy, almost the same age as him, with the same disease, but better economic status. Our other patient had coped with his disease better because of the better economic status of his family” (Participant 17).

The “lack of an integrated and purposive treatment system” included a delay in providing services to patients, inappropriate time of some nursing and therapeutic care, and lack of facilities in the ward and medication challenges. The most important concerns of the children admitted to the ward were “inappropriate time of some therapeutic and diagnostic measures or nursing cares”. A participant mentioned: “They used to wake me up at 3 in the morning for cannulation” (Participant 23).

Uninsured families or those with low-coverage insurance were in trouble paying for the treatments. A mother said, “His treatment is too expensive and there’s no one to support us. We visited State Welfare Organization and The Mahak Foundation; they said, ‘It’s a difficult-to-treat disease and not covered.’…. only pays a part of the costs of medications. We pay for the rest of the treatment costs, even though my husband is a worker” (Participant 9).

The stimulants belonging to each mode are presented in Table 2.

**Table 2:** Focal, contextual and residual stimulant in children with cancer’s adaptation to their disease

Category	Focal stimulant	Contextual stimulant	Residual stimulant
Physical challenges: Disruption in nutritional pattern	Cancer	- Unwillingness to eat at the hospital - Taste and smelling of chemotherapy medications - Inadequate ventilation in the ward - Lack of a dining hall at the ward - Serving the food on the bed	- Experience of nausea during the first course of chemotherapy - A belief that all chemotherapy medications cause nausea and vomiting
Physical challenges: Disruption in activity and rest pattern	Cancer	- Reduced playing due to physical weakness - Lack of a suitable play area at the hospital - Experience of unsuccessful play due to physical weakness	- The attitude that, due to cancer, they are not like their peers when playing - The belief that the patient should not have activity and should rest
Fragile self-concept	Cancer	- Sex - Age - passage of time - Severity of complications	- Cultural values such as high esteem for beauty - Valuing other people’s opinions too much - Poor self-confidence - Lack of independence
Difficulty of role transition	Cancer	- Age - Poor economic status - Inability to self-care	- Sense of value with continuing education and employment - Being the source of income for the family - Others’ culture
Disruption of the path to independence	Cancer	- Long-term hospitalization - Hospitalization in an isolation room - Financial burden imposed on the family - Inability to self-care - Heavy costs of treatment - Family’s economic status - Lack of all-inclusive insurance or charity coverage for treatment costs	- Significant others’ culture

## Discussion

Based on this study, children with cancer adapting to their disease did not have a steady and continuous condition, rather they had ups and downs in this area with every hospitalization or based on their disease recovery/exacerbation status and how much their social network was supportive. Based on Roy’s adaptation model, children with cancer experience numerous physical challenges in the physiological mode, especially in terms of activity, sleep and rest, nutrition, senses (pain), protection system, etc. Sibulwa et al. also declared that children with cancer experience physical challenges [2]. In the study by Kohi et al., one of the needs identified for children with cancer was a concern for physical problems [13]. These experiences impact all aspects of cancer patients’ life such as daily activities, level of independence, cognitive and physical activities, work, intimate relationships [5], quality of life, and emotional status. The ability to perform routine and daily activities is a determinant of cancer patients’ quality of life [2]. Thus, healthcare specialists should develop care measures and support strategies that assist patients’ health and functioning in daily life, both during and after the course of treatment.

The participants had a “fragile self-concept” because children’s image of their bodies was distorted. The effects of hair loss [19] and a change in children with cancer’s body image [20] lead to psychological challenges and severe alterations in self-concept [2, 21]. In the study of Negussie et al the prevalence of psychological distress among patients with cancer was high [22]. Based on the participants’ experiences, the negative impact of the loss of beauty increased as they approached puberty. Still, the consequences of hair loss were fewer for girls because they wore scarves, wigs, and headbands, and used makeup such as kohl and eyebrow pencils. Thus, it is recommended that the proper use of these covers be trained for children with hair loss, especially when they are in public.

Personal self of the participants was “Unstable”. The stress of hospitalization and diagnostic-therapeutic measures persisted like a mental obsession even when they were at home. It is known that diagnostic and therapeutic measures such as lumbar puncture and intrathecal create high levels of pain, fear, anxiety, and emotional distress in children [23]. In the present study, some children demonstrated adaptive behavior of “efforts for positive thinking”. Therefore, in addition to drug measures, nurses should extensively use storytelling, playing, jokes, and other non-drug strategies for pain management and coping with children’s emotional distress in these cases. They should also teach children relaxation methods and positive thinking to control their negative thoughts. Moreover, they should respect the patients’ religious and cultural beliefs, and seek the help of religious missionaries in the ward near the children and their families for offering spiritual support.

“Difficulty of role transition” shows the children’s efforts to adjust themselves to problems of changing roles from being a student and an offspring to a person with those roles despite cancer. They tried to adapt their responsibilities based on their physical condition, and the support of family, hospital, and school while adhering to the principles of infection control in the right condition. Continuing education was important for the children due to the public attitude and the sense of value attached to it. Chao, Chen, et al. stated that, in the Taiwanese culture, academic performance is the most important criterion for evaluating children and adolescents [24]. Thus, healthcare specialists should actively support the child’s education during treatment.

As for secondary roles, some boys aged 15–17 years old played the role of the “source of income for themselves, and the family” by working before the disease and “quitting their job after the disease” had led to the “loss of income”. Knox et al. also noted that the experience of advanced cancer disrupted developmental responsibilities [25]. Thus, it is suggested that support systems financially support these children and their families, so that financial problems would not make them delay or quit treatment.

The most important tertiary role for children was being a patient. Participants either ignored the initial symptoms of the disease themselves or spent a lot of time to confirm the diagnosis. According to Kohi et al., one of the greatest challenges identified in relation to cancer had to do with inaccurate diagnosis or treatments during the patients’ first visits, which delayed the diagnosis and onset of treatment [13]. Therefore, it is recommended that the general and specialized education for the healthcare team focus on taking the initial symptoms of pediatric cancer seriously.

Children tried to understand cancer by asking others about its nature, searching the media and the Internet, and reading books; sometimes, only the experience of living with cancer formed their understanding. Some parents tried to hide the diagnosis of cancer from their children; therefore, some participants had a vague understanding of their disease. The children themselves sometimes hid their disease to prevent pity, curiosity, and abandonment by others. After understanding the disease and a change in their body image, the children tried to hide the disease to maintain a sense of integrity and reduce harm from others. Children with cancer hope that they can be viewed as normal people and refuse to be labeled as patients [19]. Over time, they gradually get used to the diagnosis and painful and invasive treatments. Then, they talk to others about the disease or the difficult treatment becomes tolerable for them. To them, immediate hospitalization with no prior preparation and starting with painful tests on their first visit to the hospital were very unpleasant; they suggested that the child be prepared to some extent before the first hospitalization and onset of treatment. Sometimes they did not accept the limitations of the disease and showed the maladaptive behaviors in the role of the patient. Lack of adherence to treatment is a major concern in oncology because it can expose the patient to a greater risk of relapse, side effects, and poorer treatment outcomes. Adherence to treatment can affect survival, especially in types of cancer, for which chemotherapy plays a key role [26]. Results of the study by Naghavi et al. revealed that information about the nature of the disease, the side-effects of the disease, treatments, prevention of disease exacerbation, and awareness of the outcomes of lack of adherence to therapeutic recommendations, promote adherence to treatment and optimal treatment outcomes [27]. As a result, the healthcare team should prioritize measures to promote adherence to treatment. It is therefore, suggested that booklets explaining these points be developed and provided to children. Moreover, to boost the children’s spirits and, thus, promote their adherence to treatment, movies about children surviving cancer, new scientific achievements to enhance cancer treatment, and the effect of hope on recovery should be prepared and shown to the children.

Children’s social activities and interactions in exercise and religious groups were limited due to fatigue and immobility of cancer. The majority of adolescents in the study by Sibulwa et al. declared that cancer treatment and surgery negatively influence their daily activities and social interactions [2]. Thus, it seems these children should be guided about continuing their social activities without exertion while adhering to the principles of infection control.

The participants’ experiences showed a disruption of independence. Yi et al. noted that children with cancer cling to their parents because of the anxiety they experience [28]. Decker et al. also mentioned that it is essential to pay attention to the threatened independence of adolescents with cancer [29]. In today’s societies, social support is regarded as the most important facilitator of health-related behaviors; it is the most powerful method for successfully coping with stressful situations and makes it easier for people to bear difficulties [30]. Woodgate et al. stated that the need for further acceptance, attention, and care by family and friends at the time of illness are among the children’s fundamental needs after facing the disease [31]. It is, therefore, suggested that healthcare specialists prepare programs for continuing the children’s relationship with their peers and social support network virtually and in person, whether at the hospital or at home, and prepare children for continuing social interactions after discharge.

Financial support systems were insufficient. According to Kohi et al., cancer imposes an additional financial burden on the family and this concerns the child, leading to maladaptive behaviors such as quitting treatment [13]. Before assuming the role of caregiver for a child with cancer, family caregivers need to be educated and sensitized about the potential pressures they may face. Also, the formal participation of non-governmental organizations and religious institutions also ensures that family caregivers benefit from adequate community support to cope with the pressures of caregiving [32]. Thus, it is suggested that authorities and charity associations pay more attention to resolving these families’ financial problems. Support networks can positively affect the quality of life of these children and their families through financial aids and further ensuring their economic-social security. Support systems should also support children and their families in different modes.

Stimulants on each child differed from another child. Evidently, children of different age groups reacted to these stimulants differently based on their developmental age and the accessibility of regulatory and cognitive subsystems. Based on the findings, the children in the present study were at the compensatory level of adaptation; with each hospitalization and exacerbation of the disease, they made efforts to adapt to their disease by using regulatory and cognitive subsystems. Due to the difference in stimulants on each child, the existence of different subsystems, and their different support levels, these children should receive individualized care. The results of this research can be used in future studies to design intervention studies to improve the adaptation of children with cancer.

## Conclusion

Roy’s adaptation model was efficient in elucidating the experiences of children with cancer in adaptation to their disease. Children with cancer experienced almost the same physical challenges, but their experiences in the other three modes of adaptation differed depending on the stimulants as well as access to and use of subsystems. Children with influential others who reinforced their empowering behaviors and with better and more support resources were more positive and showed more adherence to treatment in adapting to their disease. On the other hand, children who were influenced by the limiting behaviors of their influential others and insufficiency of support systems considered their future as threatened. Maladaptive behaviors such as suicidal thoughts were also observed in some children. These children did not accept their role as a patient and had lower adherence to treatment. Due to the difference in stimulants on each child, the healthcare specialists, and especially nurses, should develop and implement their care and support measures in proportion to each child’s conditions to help the child adapt and safely pass the vortex of cancer.

### Electronic supplementary material

Below is the link to the electronic supplementary material.

Supplementary Material 1

## Data Availability

If someone wants to request, the data from this study should contact Dr. Fatemeh Valizadeh and Miss Fatemeh Sepahvand, who the data set used during the study is kept by them.
