# Supplementary material for: Falling and rising in the vortex of cancer: children’s adaptation with cancer: a qualitative study
Source: BMC Psychol. 2024 Apr 22;12:221. doi: 10.1186/s40359-024-01722-9 (PMC11036699; doi:10.1186/s40359-024-01722-9)
Supplement: Supplementary file 1 — Supplementary Material 1 [file 40359_2024_1722_MOESM1_ESM.docx]

**Appendix 1.** Participants’ characteristics

| Participant’s code | Participant’s role | Age | Duration of cancer/ work experience |  | Diagnosis |
| --- | --- | --- | --- | --- | --- |
| 1 | Child | 16 | 18 months |  | Hodgkin’s lymphoma |
| 2 | Child | 17 | 9 months |  | Hodgkin’s lymphoma |
| 3 | Child | 15 | 2 years |  | Acute lymphoblastic leukemia |
| 4 | Child | 9 | 5 years |  | Lung tumor |
| 5 | Child | 17 | 1 year |  | Rhabdomyosarcoma |
| 6 | Child | 6 | 1 year |  | Acute lymphoblastic leukemia |
| 7 | Child | 10 | 7 months |  | Osteosarcoma |
| 8 | Mother | 43 | - |  | - |
| 9 | Mother | 47 | - |  | - |
| 10 | Sister | 17 | - |  | - |
| 11 | Sister | 17 | - |  | - |
| 12 | Aunt | 42 | - |  | - |
| 13 | Painting teacher | 23 | 1 |  | - |
| 14 | School teacher | 38 | 1 |  | - |
| 15 | Psychologist | 30 | 2 |  | - |
| 16 | Nurse | 26 | 4 |  | - |
| 17 | Nurse | 31 | 1 |  | - |
| 18 | Nurse | 27 | 3 |  | - |
| 19 | Nurse | 30 | 3 |  | - |
| 20 | Oncologist | 43 | 12 |  | - |
| 21 | Child | 13 | 10 months |  | Osteosarcoma |
| 22 | Child | 13 | In remission, with a 2-year treatment period |  | Acute lymphoblastic leukemia |
